# Prospects of Arbuscular Mycorrhizal Fungi Utilization in Production of *Allium* Plants

**DOI:** 10.3390/plants9020279

**Published:** 2020-02-21

**Authors:** Nadezhda Golubkina, Leonid Krivenkov, Agnieszka Sekara, Viliana Vasileva, Alessio Tallarita, Gianluca Caruso

**Affiliations:** 1 Federal Scientific Center of Vegetable Production, Selectsionnaya 14 VNIISSOK, 143072 Moscow, Odintsovo, Russia; krivenkov76@mail.ru; 2 Department of Vegetable and Medicinal Plants, University of Agriculture, 31-120 Krakow, Poland; agnieszka.sekara@urk.edu.pl; 3 Institute of Forage Crops, 89 General Vladimir Vazov Str, 5802 Pleven, Bulgaria; viliana.vasileva@gmail.com; 4 Department of Agricultural Sciences, University of Naples Federico II, 80055 Portici (Naples), Italy; alessio.tallarita@yahoo.com (A.T.); gcaruso@unina.it (G.C.)

**Keywords:** onion, garlic, leek, shallot, AMF-related benefits, production, chlorophyll, antioxidants, mineral elements

## Abstract

The need to improve crop yield and quality, decrease the level of mineral fertilizers and pesticides/herbicides supply, and increase plants’ immunity are important topics of agriculture in the 21st century. In this respect, arbuscular mycorrhizal fungi (AMF) may be considered as a crucial tool in the development of a modern environmentally friendly agriculture. The efficiency of AMF application is connected to genetic peculiarities of plant and AMF species, soil characteristics and environmental factors, including biotic and abiotic stresses, temperature, and precipitation. Among vegetable crops, *Allium* species are particularly reactive to soil mycorrhiza, due to their less expanded root apparatus surface compared to most other species. Moreover, *Allium* crops are economically important and able to synthesize powerful anti-carcinogen compounds, such as selenomethyl selenocysteine and gamma-glutamyl selenomethyl selenocysteine, which highlights the importance of the present detailed discussion about the AMF use prospects to enhance *Allium* plant growth and development. This review reports the available information describing the AMF effects on the seasonal, inter-, and intra-species variations of yield, biochemical characteristics, and mineral composition of *Allium* species, with a special focus on the selenium accumulation both in ordinary conditions and under selenium supply.

## 1. Introduction

The beginning of the 21st century has been characterized by the intensive development of the arbuscular mycorrhizal fungi (AMF) market, suggesting the great economic, ecological, and nutritional impact of this innovative agronomic approach [1]. The reports devoted to AMF application [1,2,3,4,5,6,7,8] have shown the crucial role of these fungi in the development of a sustainable modern agriculture, due to ability of AMF to significantly improve soil structure, nutrient and water availability, plant tolerance to drought, high temperature, salinity, heavy metals pollution and pathogens, and the stimulation of secondary metabolites synthesis resulting in higher crop quality.

AMF are beneficial microorganisms forming symbiotic relationships with most of terrestrial plant species, and their application has become a major farming practice in modern agriculture for increasing crop yield and product quality along with a concurrent decrease of mineral fertilizers, herbicides, and insecticides utilization [9,10,11].

The efficiency of AMF utilization is connected to the AMF species, plant genotype, soil nutrient availability to plants, and environmental and stress factors [1]. *Allium* species are more sensitive to AMF application, compared to most other species, due to less developed root system [12,13,14,15,16].

*Allium* is considered one of the genera containing numerous species in plant kingdom, with more than 900 species, residing predominantly in the Northern Hemisphere. Many *Allium* representatives, such as garlic (*Allium sativum*), onion (*A. cepa*), leek (*A. porrum*), shallot (*A. cepa* L. *Aggregatum* group), chive (*A. shoenoprasum*), bunching onion (*A. fistulosum*) and others, are widely used in human nutrition [17]. Some other species are highly valuable as ornamental plants. All *Allium* species are rich in biologically active compounds, such as sulfur derivatives, quercetin, flavonoids, saponins, with significant anticancer, cardioprotective, anti-inflammation, antiobesity, antidiabetic, antioxidant, antimicrobial, neuroprotective, and immunomodulating effects [18]. Being secondary accumulators of selenium (Se), these plants express high tolerance to excess Se, synthesizing methylated forms of Se-containing amino acids and peptides, which are powerful anti-carcinogen agents [19].

*Allium* crops require high levels of N, P, and K and adequate water supply compared to other vegetables, because of weakly branched roots, causing low absorption levels of soil nutrients [20]. At the same time, high levels of nitrogen fertilizers may cause leaching, denitrification, and increase crop susceptibility to pests [21].

In this respect, the conventional practices based on massive fertilization, herbicide, and pesticide supply for production of *Allium* species [22,23] may be replaced to a large extent by the application of AMF, representing a technique for obtaining healthier products with no adverse environmental impact [9,10,24,25]. Accordingly, the exploitation of natural AMF–host potential is an important target in *Allium* crops.

The three following major factors support the AMF application for encouraging *Allium* plant growth and development:

1. high economic importance of *Allium* crops in most world countries [17,18];
2. the need to decrease the remarkable amount of mineral fertilizers and herbicides used in *Allium* crops’ production [20,26];
3. the chance to increase selenium accumulation in *Allium* plants and to produce products with high concentration of natural anti-carcinogens [19].

However, the AMF application in *Allium* crop systems is not widespread yet, despite the potential benefits from this agricultural approach, due to the insufficient knowledge at farm level about the effective interactions between AMF and plants, and the difficulties to predict the consequent advantages [27]. Besides, information about the effect of AMF on Se accumulation in plants is rather limited, the results being often contradictory for different crops including singular representatives of *Allium* species [28,29].

Table 1 demonstrates separate examples of AMF application to *Allium* species’ growth and development. The presented approaches include the utilization of individual AMF strains and their combination, and interspecies variations of response to AMF inoculation focusing on few *Allium* representatives: *A. cepa*, *A. sativum*, *A. roylei*, *A. fistulosum*, *A. porrum*, *A. galantum* and *A. cepa* L. Aggregatum group. However, the investigations report different aspects from each other, relevant to AMF effect on *Allium* crops, which hampers the appropriate comparison between the results and consequent valuable conclusions. Nevertheless, the results presented are valuable in the introduction to this new technology.

## 2. AMF Effect on Yield and Growth of *Allium* Species

Among different factors affecting the efficiency of AMF colonization [1,49,50], plant genotype and AMF species draw a special attention. Several studies have shown that most agricultural soils have a lower AMF species content compared to natural ecosystems. Furthermore, the beneficial effect of AMF to crop production with intensive fertilization is lower than that obtainable in the organic and low-input systems, due to the high soil nutrient availability (particularly phosphorus and nitrogen) in the former systems. The difference between natural and agricultural environment in AMF diversity may be connected to hyphal network disruption caused by tilling, inclusion of non-mycorrhizal species in crop rotation, use of fertilizers and fungicides, and fallow periods [51,52,53].

It is expected that large-scale production of *Allium* species entails the effect of a complex mixture of AMF species, which was confirmed in a large-scale investigation on organic and conventional onion systems in Netherlands, where 14 AMF phylotypes were identified [54]. The authors detected from one to six AMF phylotypes per field with the predominance of *Glomus mosseae–G. coronatum* and *G. caledonium–G. geosporum* species complexes, irrespective of cultivation system and sampled region [54]. The number of AMF phylotypes per field did not differ between organic and conventional farming systems. The authors demonstrated a positive correlation between onion yield and arbuscular and hyphal colonization (r = 0.70 and 0.85, respectively) under conventional management, and a lower correlation degree between arbuscular colonization and Ca content in soil (r = 0.55). AMF diversity did not affect onion yield both in organic and conventional farming.

A beneficial colonization of shallot roots by *Glomus* and *Scutellospora*, with a prevailing concentration of *Glomus*, was recorded by Priyadharsini et al. [42], whereas Mayr and Godoy [55] reported that vesicular-arbuscular mycorrhizae predominantly colonized the roots of *Allium ursinum. A. porrum* inoculation with *Glomus mosseae*, *G. intraradices*, and *G. claroideum* demonstrated the fastest roots’ colonization by *Glomus mosseae* and the lowest by *G. claroideum* [56]. On the other hand, *A. porrum* colonized by a mixture of *G. claroideum* and *G. intraradices* absorbed more P compared to the inoculation of the two single AMF. This phenomenon supposes the existence of synergism within the AMF community colonizing a single root system.

Other examples of AMF application [38,43] are consistent with the above observation. Notably, inoculation of *A. porrum* seedlings with *Rhizophagus intraradices* (RI), *Claroideoglomus claroideum* (CC), and *Funneliformis mosseae* (FM) resulted in significantly higher colonization level (59%) under the combinations RI + FM and RI + CC compared to single AMF species application. In an out-door pot-experiment on *Allium cepa*, a comparison was carried out between the effects of commercial AMF “Symbivit” preparation, containing a mixture of *Glomus* species (*G. fasciculatum*, *G. mosseae*, *G. intraradices*, *G. etunicatum*, *G. microaggregatum*, *G. claroideum*, and *G. geosporum)*, a single AMF species *(G. intraradices* BEG140) application, and a combination of ‘Symbivit’ with saprotrophic fungi (*Gymnopilus* sp, *Agrocybe praecox*, and *Marasmius androsaceus*) [38]. ‘Symbivit’ application resulted in two-fold increase of onion yield compared to control, whereas a much lower effect was recorded upon the single *G. intraradices* inoculation. Furthermore, the authors stated the existence of synergism between AMF and saprotrophic fungi causing 50% increase of *A. cepa* yield in the presence of organic matter. Both the mentioned phenomena are remarkably important for environmentally friendly production systems.

Other investigations conducted both in greenhouse pot growing [34] and in open field [32] demonstrated small differences in the beneficial effect between *G. versiforme* and *G. etunicatum* on *A. cepa* (Azar-Shahr red onion) seedlings inoculated prior to transplant. The author recorded higher AMF beneficial effect on seedlings survival (27% in field conditions), three-fold bulb yield increase, significant improvement of water use efficiency, and leaf area enhancement compared to control plants.

Interestingly, wide significant differences in AMF response may take place between various *Allium* species [40,57]. Indeed, in Chechen republic field conditions, AMF-based formulate application (Rhyzotech plus) increased plant growth and yield by 1.4 and 1.45 times in *A. cepa* and *A. sativum*, respectively [57], whereas much higher interspecies differences were reported by Galvin et al. [40] in a pot experiment. In the latter research, *G. intraradices* inoculation to *A. cepa*, *A. fistulosum*, *A. roylei*, and two hybrids *A. fistulosum x A. roylei*, RF hybrid and *A. cepa* x (*A. roylei x A. fistulosum*) led to a higher increase of plant biomass and root length in *A. fistulosum*, compared to *A. cepa*, trihybrid, *A. roylei*, and *A. fistulosum x A. roylei* [40]; the lowest effect of AMF on yield was showed by RF hybrid and the highest by *A. fistulosum.* The authors reported wide differences in AMF beneficial effect between *Allium* species or hybrids in experiments carried out in pots of different size. Furthermore, Scholten et al.’s [41] reports showed the lower AMF beneficial effect on *A. roylei* yield compared to *A. fistulosum* and *A. galanthum*.

**Figure 1:**
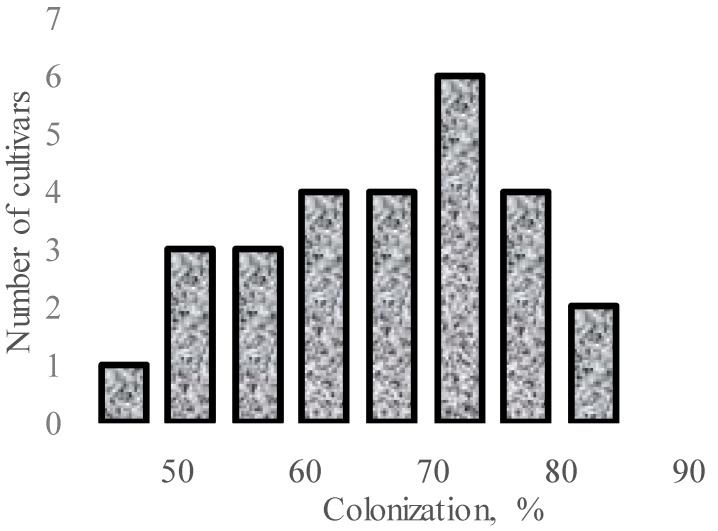
Varietal differences in *A. fistulosum* root colonization upon inoculation with *G. fasciculatus* (data re-elaborated from Tawaraya et al., 2001 [58]).

Significant varietal differences in AMF response may also take place among cultivars within *Allium* species. In this respect, investigations on 27 cultivars of *A. fistulosum* in greenhouse pot growing under *Glomus fasciculatus* inoculation revealed the significant mycorrhizal effect and the varietal differences in AMF response (Figure 1) [58]. This phenomenon was also previously recorded in other crops with better developed root systems, such as different cultivars of wheat [59], barley [60], and tomatoes [61]. Taking into account genetically determined differences of P accumulation in different cultivars, the authors suggest that the lower the ability of a cultivar to accumulate P, the higher the plant sensitivity to AMF [58].

Similar to other agricultural crops, the colonization intensity of *Allium* depends on the soil P and N contents. Studies on *Allium schoenoprasum* inoculated with *Glomus caledonium* at three levels of P and N revealed that the best crop response to this fungus was recorded at the intermediate levels of P and N, whereas the lowest fungus infection occurred under high P and N levels [62]. Previous investigation [54] reported an 18-fold increase in leaves biomass of *A. cepa* under AMF application in P-deficient soils. Moreover, the seasonal effect of AMF inoculation to *Allium* is not usually significant [33].

## 3. Effects of AMF on Physiological, Quality, and Antioxidant Indicators in *Allium* Species

The enhancement of nutrient and water efficiency upon AMF inoculation leads to physiological and quality improvement of produce. In this respect, Bolandnasar et al. [34] demonstrated 30% increase in chlorophyll content of *A. cepa* leaves under AMF application, with no significant differences between the *Glomus* species tested. The joint application of the *Glomus*-containing formulate Symbivit and saprophyte fungi to *A. cepa* resulted in a remarkable increase of bulb’s antioxidant activity (AOA) [38]. The lowest increase of onion AOA was recorded under the *G. intraradices BEG140* inoculation, where no significant differences in ascorbic acid accumulation were recorded between control and AMF-treated plants. The latter phenomenon may be connected with the low vitamin C concentration usually detected in onion bulbs. In this respect, the AOA increase in AMF-treated plants [38] is presumably related to the increase of phenolics and flavonoids, which are the main onion antioxidants, rather than to ascorbic acid. The inoculation of five different *A. cepa* cultivars with *Glomus* species (*G. versiforme*, *G. intraradices*, and *G. mosseae*) also revealed a significant increase in AOA with the highest beneficial effect caused by *G. versiforme* [30]. Field experiment in the Chechen republic (Russia) demonstrated a 32% increase in phenolics and 15% in AOA of *A. cepa* bulbs, but no significant effect on garlic antioxidant quality was found [57].

The antioxidant activity of *Allium* species is remarkably determined by the content of sulfur derivatives [63], showing high anticarcinogenic and cardioprotective effects [64]. Chemical and spectroscopic studies showed that in agricultural areas most of the soil sulfur is poorly available for plants and conversion of carbon-bonded sulfur to inorganic sulfates characterized by high assimilation levels is governed by the presence of soil microbes [65]. In this respect, AMF inoculation results in controversial effects on sulfur accumulation in *Allium* plants. Indeed, in a pot experiment mycorrhizal colonization of *A. cepa* roots with *Glomus versiforme* or *Glomus intraradices* BEG141 did not significantly affect bulb enzyme-produced pyruvate or sulfur accumulation levels in shoots, and a higher yield and N and P accumulation levels increase was elicited by *G. versiforme* inoculation [66]. Another investigation regarding N and S supply to *A. fistulosum* revealed that total and organic sulfur content in shoots and enzyme-produced pyruvate were significantly lower in case of *Glomus mosseae* utilization compared to control plants [67]; conversely, the same indicators attained higher or not different values in plants treated with *Glomus intraradices* compared to control. Moreover, *Glomus fasciculatum* application increased alliin content and alliinase activity in garlic [47].

**Figure 2:**
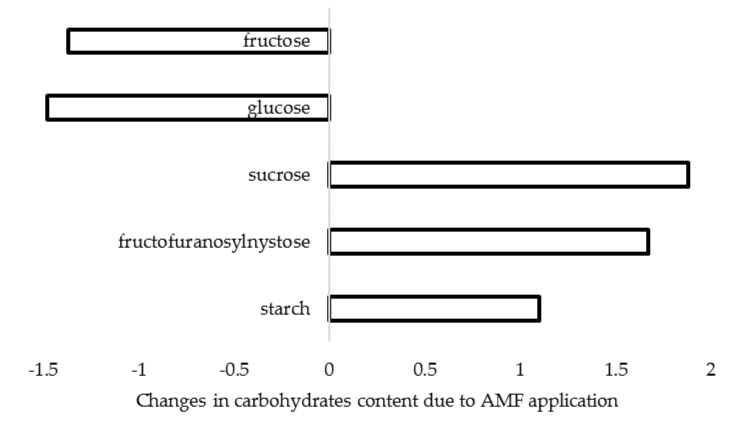
Changes in carbohydrate content in *A. cepa* bulbs as a result of *G. intraradices* and *G. mosseae* inoculation (data re-elaborated from Lone et al., 2015 [71]).

Salvioli et al. [68] reported that mycorrhizal colonization significantly impacts host gene expression and metabolomic profiles. Indeed, AMF inoculation is known to affect photosynthesis and the sugar metabolism [69], for instance, via stimulation of biosynthesis of phytohormones, such as abscisic acid [70]. In this respect, *Glomus fasciculatum* and *G. mosseae* application to *A. sativum* resulted in increased chlorophyll and sugar content in leaves [28]. Furthermore, Lone et al. [71] recorded significant changes in carbohydrates content of *A. cepa* bulbs treated with *G. intraradices* and *G. mosseae*, and in particular the decrease of monosaccharides (reducing sugars) and the increase of disaccharides and injectable oligosaccharides (Figure 2). Conversely, a 68% increase of monosaccharides content in *A. cepa* inoculated with mycorrhizal formulate Rhizotech Plus was reported in a field experiment [57].

**Figure 3:**
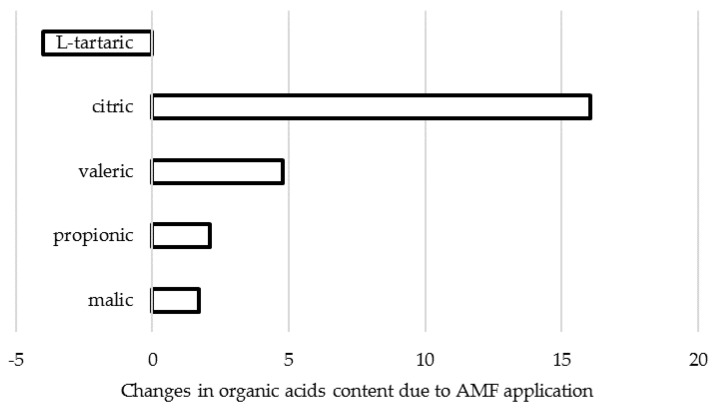
Changes in organic acids content in *A. cepa* cultivar Wolska upon AMF (*Rhizophagus irregularis*) inoculation (data re-elaborated from Rozpḁdek et al., 2016 [74]).

The biosynthesis of organic acids and sugar metabolism are known to be closely related. Indeed, titratable acidity (TA) increase upon AMF application was recorded in tomato [72]; in shallot [11] and onion [57,73] under open field double inoculation, with a 69% and 162% increase, respectively; in open field *A. cepa* treated with “Rhizotech Plus” formulate, with a 45% rise [57]. In research carried out in Poland, Rospadec et al. [74] reported the TA increase in *A. cepa* bulbs under *Rhizophagus irregularis* application (Figure 3), with significant changes in organic acids content from: malic > propionic > tartaric > valeric > citric before inoculation, to malic > propionic > citric > valeric > tartaric after inoculation.

Organic acids are synthesized via oxidation of photosynthetic assimilates. Their biological functions include production of amino acids and ATP, maintenance of redox balance and membranes permeability, and acidification of extracellular spaces. Organic acids release by roots result in soil acidification, thus improving plant nutritional efficiency, including either phosphorous and iron accumulation or their transport across xylem [73]. The data shown in Figure 3 suggest the increase of total organic acid pool including malate and in particular, citrate in *A. cepa* bulbs under AMF application. The latter phenomenon may also be connected with the increase in nitrogen accumulation and has a beneficial effect on plant development, thanks to the participation of organic acids in energy and amino acids production.

## 4. Effects of AMF on Elemental Composition

AMF inoculation reportedly increases the accumulation of phosphorous, potassium, nitrogen, and several essential microelements, as a consequence of root system enhancement. Increase in N, P, and K content was detected in *A. cepa* upon the inoculation of AMF (*Glomus sp. + Gigaspora*) and sulfur oxidizing bacteria [36]. Inoculation of *A. cepa* plants with *Glomus versiforme* resulted in higher accumulation of N, P, and Zn compared to plants treated with *Rhizophagus intraradices* [75,76]. The mycorrhizal infected plants of *A. cepa* also showed a higher efficiency in nutrient and water uptake [77] and enhanced adaptation to different water accessibility in the soil [78]. According to Bolandnazar et al. [34], *Glomus versiforme* significantly enhanced the water use of *A. cepa* under water-deficit conditions [79]. Accordingly, the AMF-inoculated *A. cepa* plants demonstrated higher evapotranspiration, increased leaf area, biomass, and higher yield compared to control [21,34]. Investigations on *A. cepa*, cultivar “White Lisbon” [80] and *A. cepa* cv. Alfa São Francisco—Cycle VIII (Embrapa Semi-Árido) [37] demonstrated that joint application of humic substances and AMF (*Glomus intraradices* [80] and *Rhizophagus intraradices* [37]) synergistically increased growth and nitrogen and phosphorous levels [37,80], as well as protein content [37]. The interaction between humic substances and AMF also resulted in the highest accumulation of sugars and proteins in leaves, and consequently caused higher yield and quality of onion bulbs [37]. Other works indicate that AMF are able to secrete acid phosphatases, thus improving P accumulation [81]. AMF may affect the metals’ utilization by plants via element immobilization on their hyphae cell walls, chelating them by glomalin or via compartmentalization inside cells. Testing three commercial AMF formulates “Pla” (TerraVital Hortimex + *G. mosseae*, *G. intraradices + G. claroideum + G. microaggregatum*), “Bio” (Endorize-Mix + *G. mosseae*, *G. intraradices*), and “Tri” (*G. mosseae + G. intraradices + G. etunicatum*) in pot experiment with peat substrate containing from 20% to 40% of compost revealed significant increase of potassium and Zn content in *A. porrum* pseudostems compared to control plants. In the latter conditions, AMF colonization did not affect plant growth and P uptake, supposedly due to the lower availability of P organic forms to some AM fungi compared to inorganic P forms [46].

Expression of plant tolerance genes to different metals is known to greatly depend on the intensity of mycorrhizal colonization [5]. In this respect, a special attention should be focused to AMF effect on the accumulation of selenium, which is not considered an essential element for plant growth and development, though it shows significant antioxidant protective effects [66] and is a natural analog of sulfur, often replacing the latter element in biological systems [82].

## 5. Selenium–AMF Interaction

All *Allium* species belong to secondary selenium accumulators and are characterized by the ability to convert inorganic forms of soil selenium to methylated forms of Se-containing amino acids, such as selenomethyl selenocysteine and selenomethyl selenomethionine. This prevents the incorporation of such amino acids into proteins, contrary to plants non-accumulators where no methylated forms are synthesized and the transport of selenium to proteins causes the decrease of enzyme’s biological activity and accordingly toxicity [83]. Moreover, methylated Se containing amino acids are highly valuable for human health, thanks to their pronounced anti-carcinogenic properties [84,85]. In particular, the Se-analogs of the sulfur compounds in *Allium*, such as diallyl selenide and benzyl selenocyanate, were reportedly more effective as anticarcinogenic agents than diallyl sulfide and benzyl thiocyanate [86].

Up to date, a lot of investigations have been devoted to plant biofortification with Se, leading to products with high antioxidant activity and able to treat Se deficiency, which affects about 15% of the world population due to Se poor soils [87]. At the same time, plant enrichment with Se entails a serious ecological problem, taking into account that either soil or foliar Se supply shows low efficiency, thus resulting in a remarkable amount of unabsorbed Se with consequent environmental pollution; in addition, a significant fraction of soil Se is poorly available to plants.

Currently, plenty of papers report the involvement of soil microorganisms in redox biotransformation of Se [88], which causes the protection of plants against high concentrations of Se, thus improving the tolerance to selenium accumulation. However, the findings are controversial, as Patharajan and Raaman [28] suggested the depression of AMF sporulation in soil under selenium dioxide supply, whereas Larsen et al. [29] indicated the opposite effect on sodium selenate. Results on the effects of AMF inoculation on other species’ growth and development under Se supply are also controversial. Indeed, Duràn et al. [89] showed 23% increase in Se content in wheat upon the joint inoculation of *Glomus claroideum* and bacteria tolerant to Se (*Stenotrophomona* sp. B19, *Enterobacter* sp. B16, *Bacillus* sp. R12, and *Pseudomonas* sp. R8). On the other hand, AMF inoculation of several other agricultural crops, such as lettuce [90], maize, alfalfa, and soybean [91], reduced the Se content in plants. Similar controversies arose from studies focusing on AMF effect on accumulation of sulfur, which is a Se chemical analog [47,66,67,92].

Scant literature is available about the effect of AMF on the efficiency of Se biofortification in plants, especially in *Allium* species, as well as regarding the effect of different AMF species on the intensity of Se biofortification in *Allium* crops. Nevertheless, previous investigations suggest the interesting prospects of AMF utilization for improving *Allium* Se status. In this respect, Larsen et al. [29] demonstrated the chance of increasing garlic selenium content through soil inoculation with *Glomus intraradices* and of improving Se biofortification by joint application of AMF and sodium selenate, which enhances Se content from 1.5 to 15 mg·kg^−1^ d.w. The authors reported that the predominant form of Se in AMF + Se treated *A. sativum* was γ-glutamyl-Se-methyl-selenocystein, which represents more than two-thirds of total Se and is the compound with the highest anticarcinogenic effect [29].

A significant Se-AMF interaction was detected in shallot plants inoculated with *Glomus*-based formulate (“Rhisotech plus”) under organic (selenocystine) and inorganic (sodium selenate) Se supply [11]. The results suggest that AMF inoculation increased soil Se bioavailability in shallot plants by up to 8 times. A lower beneficial effect of AMF application was recorded under both organic and inorganic Se supply, i.e., the Se biofortification levels were increased by 5.9 and 4.4 times, respectively, compared to control plants. The organic Se form resulted in higher Se biofortification level than sodium selenate, both with and without AMF application [11].

The AMF beneficial effect on Se accumulation was also demonstrated in *A. cepa* and *A. sativum* grown in the same environmental conditions [57] (Table 2). Higher Se concentrations in fortified garlic bulbs grown with or without AMF inoculation reflect the higher ability of *A. sativum* to accumulate sulfur compared to *A. cepa* [93,94,95], which is consistent with the report that *A. sativum* is a more powerful natural anti-carcinogen than onion [85].

Overall, in the absence of exogenous Se, “Rhyzotech plus” AMF-based formulate inoculation increased Se content by 5 times in garlic, 10 times in onion, and 8 times in shallot bulbs [11,57]. Notably, contrary to *A. sativum and A. cepa*, shallot accumulated significantly lower levels of Se in conditions of joint Se and AMF application (not more than 5000 µg·kg^−1^ d.w.). In this respect, the results suggest that the efficiency of joint AMF + Se application on Se accumulation in the mentioned *Allium* representatives depended on the species and was the highest in *A. sativum* and decreased in *A. cepa* and shallot bulbs.

Interestingly, the effective AMF Se-fortification of garlic and onion leads to high prospects for producing functional food with the ability to significantly enhance the human Se status and, at the same time, to provide compounds with high anticarcinogen activity. According to Golubkina et al.’s [57] reports, 5 g of Se-fortified fresh garlic bulbs ensure 50% of the adequate Se consumption level (ACL is 70 mcg·day^−1^), whereas 50 g of Se-enriched onion bulbs will give as much as 1.4 ACL for Se.

Another aspect of *Allium* species biofortification with Se under AMF inoculation is the effect of joint Se-AMF application on biochemical characteristics and mineral content of the produce. Lacking literature reports for most crops makes these investigations extremely attractive. In this respect, the studies carried out on shallot, onion, and garlic under AMF-based formulate application revealed the following details [11,57]: the AMF beneficial effect on yield, carbohydrates, TA, and antioxidant content in the mentioned *Allium* species was enhanced by Se supply (Table 2). The data presented in Table 2 suggest that the joint Se–AMF application showed a higher beneficial effect than AMF inoculation on yield, monosaccharides, flavonoids, ascorbic acid, and AOA levels of *A. cepa*, the latter species attaining higher values compared to *A. sativum*. Moreover, among the antioxidants analyzed, an ascorbic acid increase in shallot bulbs [11] and *A. sativum* and *A. cepa* [57] was recorded in plants treated with AMF or sodium selenate (by 1.3 times compared to control plants in shallot bulbs [11], and lower levels in garlic and onion [57]; Table 2). Differently, the increase in flavonoids content was detected both in shallot plants inoculated with AMF or treated with AMF + selenocystine (by 1.44 and 1.33 times, respectively), whereas the highest increase in flavonoids content under AMF + Se application (1.7 times compared to the untreated control) was recorded in *A. cepa* bulbs. Notably, phenolics were not affected by the joint application of AMF and Se in *A. sativum* and just slightly in *A. cepa*.

Even wider differences between the effect of AMF and AMF + Se application were recorded for macro- and trace elements accumulation. Indeed, joint Se–AMF utilization stimulated the accumulation of K in onion and garlic bulbs and of Mg in onion. A phosphorus increase was detected only in garlic, whereas Ca content was much higher in plants inoculated with AMF without Se supply. Moreover, under Se + AMF application, a remarkable increase of B, Fe, Zn, and Si concentration was detected in *A. cepa* bulbs and of Zn and Fe in *A. sativum*.

The observed differences in sodium concentration due to AMF–Se applications (Table 2) may be attributed to the higher tolerance to salt stress of *A. sativum* compared to *A. cepa* [96] and the known relationship between Se and Na in plants [11].

One of the most significant effects of Se–AMF interaction seems to be the intensive increase in Mo accumulation both in *A. cepa* and *A. sativum*, which has never been reported previously. Notably, this element participates in plants redox reactions via Mo-containing enzymes including sulfite oxidase, xanthine dehydrogenase, nitrate reductase, and aldehyde oxidase, thus particularly taking part in nitrogen metabolism and phytohormones synthesis, including abscisic acid and indole-3 butylic acid. The latter fact may be of special importance as one of the possible mechanisms of *Allium* plants’ growth stimulation under joint application of Se and AMF.

Notably, results stemmed from investigations on shallot did not fully confirm the data presented in Table 2. In fact, the application of AMF-based formulate to shallot plants led to the increase of B (64.7%), Cu (47.4%), Fe (141%), Mn (77.9%), Zn (44.7%), and Si (78.4%) [11], whereas no differences in Cu and Mn accumulation between AMF and AMF + Se-treated *A. sativum* bulbs were recorded. Furthermore, shallot plants inoculated with AMF showed a reduced ability to accumulate Al, and this phenomenon was not reported for *A. sativum* and *A. cepa* [57]. The mentioned data suggest that the joint Se + AMF application may either not differ from that of AMF inoculation or even be more effective than the latter.

## 6. Conclusions

From the reports of the present review, three important aspects should be highlighted: (i) the inoculation of AMF species consortia usually provides higher beneficial effects than the single AMF species application, thus showing interesting utilization prospects within *Allium* crop systems; (ii) AMF application may become a new approach for producing functional food with high anticarcinogen activity, in interaction with Se biofortification of *Allium* species; (iii) the effect of AMF inoculation on mineral composition of *Allium* plants is species-dependent, and we have not found literature reports revealing these peculiarities, except for those relevant to N and P.

In general, the application of AMF to *Allium* species commonly grown as vegetables leads to significant enhancement of yield, physiological and quality indicators, antioxidant compounds and activity, and mineral content, in particular, selenium concentration. However, the effect of AMF on the mentioned crops greatly depends on plant genotype, AMF single species or species consortium, farming management, and soil and environmental conditions.

## Figures and Tables

**Table 1:** Arbuscular mycorrhizal fungi (AMF) application on *Allium* species.

AMF	Effect	References
***Allium cepa***		
*Glomus versiforme*	Increase in antioxidant activity (AOA). Intervarietal differences in red, pink, yellow, and white *A. cepa* varieties.	[30]
*G. mosseae*, *G. soronatum*, *G. caledonium*, *G. geosporum*	The highest crop yield under *G. mosseae* and *G. caledonium* application.	[31]
*G. versiforme*, *G. intraradices*, *G. etunicatum*	Enhancement of seedling growth, mineral element, water use efficiency, and 3 times yield increase (>35 t/ha). Highest increase of leaves’ surface promoted by *G. versiforme*.	[32]
*G. mosseae*	Field and greenhouse results: significant yield and growth increase both in winter and autumn.	[33]
*G. versiforme*, *G. intraradices*, *G. etunicatum*	*G. etonicatum* gave the highest increase in bulb caliber, biomass, leaf surface, dry matter, chlorophyll content.	[34]
*G. fasciculatum*	Stimulation of nitrate reductase and glutathione synthetase activity in shoots and roots under water stress.	[35]
AMF *+ Thiobacillus* sp.	High concentrations of N, P, K, and S in soil rhizosphere at 60 and 90 days from planting.	[36]
*Rhizophagus intraradices* + humic substances + CO_2_	Enhancement of growth and quality of onion seedlings, chlorophyll, soluble solids, proteins, and proline content in leaves.	[37]
*AMF mixture of Glomus etunicatum*, *G. microaggregatum*, *G. intraradices*, *G. claroideum*, *G. mosseae*, *and G. geosporum*; single-fungus inoculum of *G. intraradices BEG140*; bark chips pre-inoculated with saprotrophic fungi mixture: *Gymnopilus* sp., *Agrocybe praecox + Marasmius androsaceus*	Higher beneficial effect of joint AMF application compared to a single-fungus; a synergism between AMF and saprotrophic fungi.	[38]
AMF	AMF reduces soil pH more than non-inoculated plants in the presence of NH_4_.	[39]
***Allium cepa*, *Allium roylei*, *Allium fistulosum*, *A. fistulosum×A. roylei* hybrid**		
*Glomus intraradices*	Interspecies differences in response to AMF inoculation.	[40]
***Allium fistulosum*, *Allium roylei*, *Allium galanthum***		
*G. mosseae*, *G. intraradices*	Interspecies differences to AMF response. No differences between the effects of the two AMF species.	[41]
***Allium cepa* L. var. *aggregatum***		
AMF and dark septate fungal endophyte (DSE) associations	Significant correlations between soil P and microsclerotia and between soil N, K and AMF spore number.	[42]
Rhizotech MB *formulate:* *Glomus intraradices* with low concentrations of *Trichoderma harzianum* and *Bacillus subtilis*	Increase in Se accumulation.	[11]
***Allium porrum***		
*Rhizophagus intraradices* (RI), *Claroideoglomus claroideum* (CC), *Funneliformis mosseae* (FM)	Highest colonization with (RI + FM) and (RI + CC).	[43]
*Funneliformis mosseae*, *Claroideoglomus claroideum*, *Rhizophagus intraradices*	AMF decreases nitrate leaching.	[44]
AMF + biochar	Protection from heavy metals. Biochar decreases colonization degree of AMF.	[45]
AMF + compost + peat	Increase of leaf Zn and K, but no effect on dry matter, N, and P.	[46]
***Allium sativum***		
*G. mosseaeG. fasciculatum* + SeO_2_	The highest beneficial effect of *G. fasciculatum* application:decreases of AMF colonization degree due to SeO_2_ application	[28]
*G. fasciculatum*	Increase in alliin content and alliinase activity.	[47]
*G. fasciculatum +* P	Highest colonization and yield at 20 kg·ha^−1^ P.	[48]
*Glomus intraradices*	Increase of selenate accumulation.	[29]

**Table 2:** Effect of AMF–Se application on biochemical parameters and elemental composition of *A. sativum* and *A. cepa* (% to control plants) (data re-elaborated from Golubkina et al., 2020 [57]).

Parameter	AMF		AMF + Se		Se	
	*A. sativum*	*A. cepa*	*A. sativum*	*A. cepa*	*A. sativum*	*A. cepa*
Yield	149	145	156	150	100	100
Dry matter	108	119	104	119	109	100
monosaccharides	100	168	175	200	129	141
Total sugar	119	100	121	100	119	100
AOA	100	115	108	137	100	100
Flavonoids	100	100	118	169	100	100
Ascorbic acid	100	100	110	124	100	112
TA	88	145	118	111	100	131
Minerals						
Se	488	1000	28,200	43,800	21,300	33,200
P	110	130	122	130	110	100
K	100	123	123	140	112	100
Ca	145	155	100	100	100	100
Mg	191	100	186	123	191	100
Na	206	100	141	100	168	129
B	100	108	100	126	100	126
Fe	100	131	116	236	89	100
Cu	100	143	100	135	100	100
Mo	140	60	284	144	216	100
Zn	100	100	142	154	125	100
Si	68	108	100	152	100	132
